# Automated nucleic acid chain tracing in real time

**DOI:** 10.1107/S2052252514019290

**Published:** 2014-09-23

**Authors:** Kevin Cowtan

**Affiliations:** a Department of Chemistry, University of York, York YO1 5DD, England

**Keywords:** nucleic acid chain tracing, *Coot*

## Abstract

The crystallographic structure solution of nucleotides and nucleotide complexes is now commonplace. The resulting electron-density maps are often poorer than for proteins, and as a result interpretation in terms of an atomic model can require significant effort, particularly in the case of large structures. While model building can be performed automatically, as with proteins, the process is time-consuming, taking minutes to days depending on the software and the size of the structure. A method is presented for the automatic building of nucleotide chains into electron density which is fast enough to be used in interactive model-building software, with extended chain fragments built around the current view position in a fraction of a second. The speed of the method arises from the determination of the ‘fingerprint’ of the sugar and phosphate groups in terms of conserved high-density and low-density features, coupled with a highly efficient scoring algorithm. Use cases include the rapid evaluation of an initial electron-density map, addition of nucleotide fragments to prebuilt protein structures, and in favourable cases the completion of the structure while automated model-building software is still running. The method has been incorporated into the *Coot* software package.

## Abstract 2

A method is presented for the automatic building of nucleotide chains into electron density which is fast enough to be used in interactive model-building software. Likely nucleotides lying in the vicinity of the current view are located and then grown into connected chains in a fraction of a second. When this development is combined with existing tools, assisted manual model building is as simple as or simpler than for proteins.

## 1. Background   

The structural analysis of RNA and DNA molecules and complexes has become important in recent years, with the PDB now reporting nearly 3000 deposited structures containing nucleotides (Berman *et al.*, 2007 ▶). The interpretation of nucleotide electron-density maps presents different challenges to the interpretation of protein maps: the resolution of the data is frequently lower (Keating & Pyle, 2010 ▶), the monomers are larger and therefore more flexible, and in some cases the structures are very large (see, for example, Wimberly *et al.*, 2000 ▶). While graphical software such as *Coot* (Emsley *et al.*, 2010 ▶) can be used for building nucleotides, the tools available are less mature than for proteins. A new approach is described which allows interactive backbone tracing of nucleotides in *Coot*, which may be used in combination with existing tools such as *RCrane* (Keating & Pyle, 2012 ▶) to produce a complete and accurate model.

There are a number of existing packages for automated or semi-automated nucleotide building. The *RESOLVE* software, now incorporated in *phenix.autobuild*, will build nucleotide chains in a fully automated manner when the data are of sufficient quality (Terwilliger *et al.*, 2008 ▶). The *ARP*/*wARP* package will also build nucleotide chains, but not assign the base types (Hattne & Lamzin, 2008 ▶). The *LAFIRE* package includes software for extending and rebuilding existing nucleotide chains (Yamashita *et al.*, 2013 ▶). The *RCrane* software performs semi-automated building, in which the software aids the user in the location of the ribose and phosphate groups and then builds a chain through the resulting groups, and can be used interactively from within the *Coot* graphical model-building software.

The methods described here are complementary to the *RCrane* development as they provide a method for the instantaneous building of segments of nucleotide chain in the region of an electron-density map currently being viewed in the *Coot* program. These segments may then be corrected and rebuilt automatically using the *RCrane* tools. The primary design consideration for the method was speed, enabling the user to build tens or even hundreds of nucleotides in a few minutes. For interactive use the software must therefore be able to find a nucleotide chain in the general vicinity of the current view position and trace it in both directions as far as the density supports in a fraction of a second. This is achieved through a carefully designed search function which can rapidly score electron density according to the likely presence of a nucleotide feature at that position.

## 2. Methods   

The nucleotide chain-tracing algorithm has two fundamental stages: (i) finding nucleotides as a starting point for building and (ii) growing extended nucleotide chains. Finding nucleotides is performed using an optimized search algorithm and a search ‘target’ describing the electron-density feature to be recognized; these will be described in §[Sec sec2.1]2.1. Growing the nucleotides into chains involves converting the search targets into single-nucleotide or binucleotide fragments and then extending these fragments by adding additional nucleotides using a database of chain conformations; these steps are described in §[Sec sec2.2]2.2.

### 2.1. Finding nucleotides as a starting point for building   

#### 2.1.1. Fragment-search algorithm   

In order to achieve fully interactive performance, careful consideration must be given to the search algorithm. The location of three-dimensional model fragments usually requires a six-dimensional search over three positional and three orientation parameters. While it is possible to factor out the orientation search by searching for spherically averaged density (Vagin & Isupov, 2001 ▶), this involves discarding information about the shape of the fragment. The alternative is to search over all three orientation parameters and then use a highly optimized positional search.

One approach to optimizing the positional search is to make use of fast Fourier transforms (FFTs), as employed in molecular-replacement calculations (Navaza & Vernoslova, 1995 ▶) and related methods (Cowtan, 2001 ▶). However, a three-dimensional FFT still requires of the order of 3log(*n*) computations per grid cell, where *n* is the number of grid cells along one edge of the unit cell. FFT-based methods also tend to be calculated over the entire unit cell or at least the asymmetric unit (Read & Schierbeek, 1988 ▶), although it is possible to relax this constraint. When working interactively the user often wants to build the electron-density feature that they are currently viewing (and continuations of this feature in the case of long polymers); this provides an additional optimization which can be exploited by non-FFT methods. Optimal performance is then achieved by minimizing the average number of computational operations required per grid cell to identify the presence of a model fragment in a given orientation at that position.

Kleywegt & Jones (1997 ▶) developed a method for locating the presence of molecular fragments using a scoring metric based on the electron density at the atomic centres of the oriented fragment after translation to the current grid position. However, this approach has the limitation that a large ‘blob’ of high density which is larger than the fragment will score highly even if it does not resemble the fragment in shape. In the FFT-based method of Cowtan (2001 ▶) it was found that better fragment discrimination could be obtained by using a scoring function which requires both high density where there are expected to be atoms and low density where there are expected to be none.

The latter approach may be adapted for use in a fast non-FFT-based approach by defining a set of probe points describing the ‘fingerprint’ of the search fragment in terms of a set of positions where the electron density must be high if the oriented fragment is present and a second set of positions where the electron density must be low if the fragment is present: these will be referred to as high and low probe points, respectively. A scoring function is then used to evaluate the electron densities at the high and low probe points and return a single value to indicate the presence or absence of the fragment.

Let the positions of the high probe points relative to the centre of the oriented fragment be δ*x* ^h^, the positions of the low probe points relative to the centre of the fragment be δ*x* ^l^ and *n* be the number of probe points of each type. (For simplicity of implementation the numbers of high and low probe points are constrained to be equal.) A simple score may be obtained from the difference between the means of the densities at the high and low probe points, 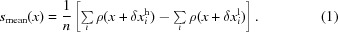
However, this scoring function does not meet the criteria for very fast evaluation since it must be evaluated for every probe point. To reduce the computation to an average of a few operations per grid cell a different form is adopted, 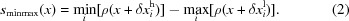
The advantage of this form is that in most cases only part of the expression needs to be evaluated. Since the minimum can only decrease and the maximum can only increase with the inclusion of further probe points, the score can only become worse as it is evaluated.

Evaluation therefore takes place one pair of probe points at a time, and as soon as the partial score drops below a given threshold, evaluation is terminated and the existence of the oriented fragment at that position is rejected. The score threshold may start at zero (implying that for a fragment to be accepted the lowest high probe must be greater than the highest low probe), although other values are possible.

Three further optimizations are employed.

(i) The positional offsets are rounded to the nearest grid vector, so that the electron density is evaluated at a grid point rather than by interpolation between grid points.
(ii) A target number of matches is chosen and the score threshold is periodically adjusted upwards to retain no more than the target number of matches and allow the earlier termination of further scoring steps.
(iii) Fragment translations where the density at the first high probe point is less than one standard deviation above the mean are eliminated immediately.

Rotation space is explored with an 18° search step, giving a total of 2792 orientations. The number of translations within 6 Å of the view centre depends on the grid resolution, but is typically around a thousand. Pruning based on the first high probe point typically reduces this number by a factor of six. Initially, a little under half of the remaining points are pruned as each new pair of probe points is included in *s*_minmax_; however, as the calculation progresses and the score threshold is raised more translations are subject to early pruning.

The approximations mean that the calculation is crude but very efficient. In practice, this scoring method is used to provide a list of candidate fragment locations which may then be re-evaluated using the more sensitive *s*_mean_ score using interpolated electron-density values. The calculation is repeated for each possible orientation of the fragment by a search over the three rotational parameters.

#### 2.1.2. Search targets   

Each nucleic acid monomer provides three rigid groups which may be used as search fragments: the pentose sugar, the phosphate group and the base. The sugar and phosphate groups are used for identifying the main chain: this is in contrast to the method of Hattne & Lamzin (2008 ▶), in which the base is located first. For each of these search fragments a set of high and low probe points must be identified.

The probe points were determined using the electron density of nucleotides from the structure with PDB code 1hr2 (Juneau *et al.*, 2001 ▶): a total of 316 nucleotides, with the conserved atoms of the search fragment superimposed. Less common conformations of the atoms of the search target were removed by successively removing fragments in which the O2′ atom (for sugars) or C5′ and C3′ atoms (for phosphates) were more than 1 Å from the ensemble mean for that fragment, leaving 280 sugar fragments and 226 phosphate fragments. An electron-density map for the structure was calculated from the observed data at 2.2 Å resolution. The maximum and minimum across all of the fragments in this map for each grid point around the oriented fragment were determined and used to calculate a minimum and maximum map.

For every grid point in the minimum map, every instance of the fragment has an electron-density value greater than or equal to the value at that position in the minimum map. As a result, a high value in the minimum map is a strong indication of a conserved electron-density feature which is consistent with the presence of the fragment. Similarly, a low value in the maximum map corresponds to a position where no instance of the fragment has high electron density, which indicates a conserved electron-density hole consistent with the presence of the fragment. The minimum and maximum maps may therefore be used to locate high and low probe points, respectively.

High probe points are placed on atoms of the search fragment (O3′, C1′, C2′, C3′, C4′, O4′ and C5′ for the sugar, and O3′, P, OP1, OP2 and O5′ for the phosphate), omitting atoms which are not strongly conserved (*e.g.* O2′). Additional probe points are allocated to represent density not consistently associated with a single atom; thus, for example, in the case of sugar fragments the base is represented by a probe point on the N1 or N9 atom and a second point at the far side of the associated ring, but not on an atomic centre.

Low probe points are dispersed around the perimeter of the fragment. The contour level of the maximum map was set to the lowest value in the map within a 6 Å radius of the fragment centre, and a low probe was placed at this point. The contour level was then gradually increased and probe points were placed in new density features as they appeared, subject to the constraint that a new probe point should not be too close to an existing probe point so as to provide independent information.

**Figure 1:**
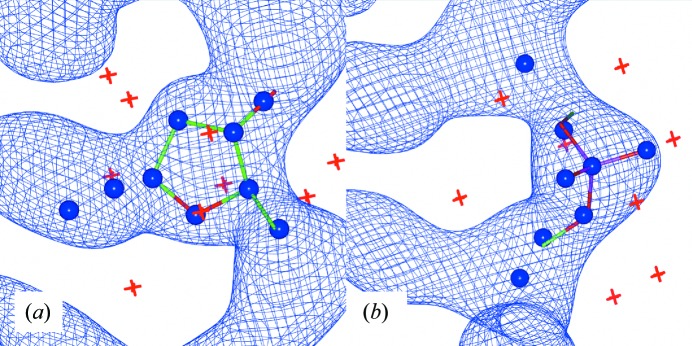
High and low probe points (spheres and crosses, respectively) for (*a*) the sugar target and (*b*) the phosphate target. The average electron density over the reference set of nucleotides is shown.

The minimum and maximum maps, and the corresponding high and low probe points, are shown for each of the two search fragments in Fig. 1 ▶. The coordinates of the probe points are provided as Supporting Information.

### 2.2. Growing an extended nucleotide chain   

#### 2.2.1. Converting the initial fragments to nucleotides   

The initial candidate fragments are then grown into extended chain fragments. Candidate fragments which fail to grow to at least three nucleotides are rejected. This process comprises two steps. Firstly, the initial fragments must be extended to complete single nucleotides or binucleotides. Next, they are iteratively extended using an algorithm which successively adds additional nucleotides to either the 3′ or the 5′ end of the chain.

Both of these processes involve a database of nucleotide chain linkages, which are determined from one (or potentially more than one) known high-quality nucleotide structure. The database consists of fragments of connected nucleotides, in which each nucleotide is represented by the main-chain atoms. The key functionality of the database is to provide fragments matching one or more sugar rings (or phosphate groups). If a single sugar ring is provided, then a list of short chain fragments are returned with every sugar ring from the database superposed on the given sugar. If multiple sugar rings are provided then every possible chain fragment which is capable of linking those sugars is returned.

The default database is also constructed using the structure with PDB code 1hr2 (Juneau *et al.*, 2001 ▶) and thus contains 316 nucleotides. Limiting the size of the database improves performance and has also been found to reduce the chance of the incorporation of incorrect rare conformations when data quality is poor. When the data are good a larger database can sometimes improve the results if interactive performance is not required.

Since the database relies upon oriented sugar rings for further building, the sugar rings located in the initial search may be used directly as a starting point for chain tracing. Phosphate groups, however, require an additional step. Every phosphate group from the database is superposed on the group identified from the electron density. The two neighbouring sugars are then scored against the density using *s*_mean_ and the best-scoring binucleotide is stored as a starting point for chain tracing. The result of this step is a set of mononucleotides (from the location of sugar rings) and binucleotides (from the location of phosphate groups); however, in each case the terminal phosphate group is in an arbitrary conformation so that the fitted parts of the fragments actually correspond to nucleosides and ‘suites’, respectively (Murray *et al.*, 2003 ▶).

The use of known structure fragments to describe the range of possible main-chain conformations is in contrast to the approach of Keating & Pyle (2010 ▶), who use the database of distinct sugar–sugar backbone conformers (or ‘suites’) determined by Murray *et al.* (2003 ▶). The latter approach is probably more efficient because redundant conformers are eliminated, and will be explored in future; however, the known structure database can also be used to bridge multi-nucleotide gaps using a method analogous to loop fitting in proteins (Cowtan, 2012 ▶).

#### 2.2.2. Growing chains   

Chains are then grown from the initial fragments by adding further nucleotides at either end. Each sugar ring from the database is superposed on the 3′ or 5′ sugar of a fragment, and the next (or previous) nucleotide from the database is extracted and scored against the map using the *s*_mean_ score for both the new sugar and the intervening phosphate group. Building continues at each end of the chain until no sugar ring can be found whose score exceeds a threshold value. The threshold is determined by scoring 100 000 random translations and orientations of the search target in the current electron-density map to establish a probability distribution of score values. The threshold score is set such that the probability of a score exceeding this value is 0.1%.

The algorithm as described can often produce multiple overlapped chain traces starting from different initial fragments. Merging of these fragments and resolving branches is achieved using the method described for proteins in Cowtan (2006 ▶).

The sugar puckers are often ambiguous in the electron density, and the method as implemented does not attempt to resolve sugar puckers except to the extent that the sugar pucker is implicit in the sugar–phosphate–sugar backbone conformation. The effectiveness of this approach has not been investigated and therefore post-refinement using *RCrane* (Keating & Pyle, 2012 ▶) is advisable.

## 3. Evaluation   

### 3.1. Search targets   

The skill of the search target functions in locating sugar and phosphate groups was evaluated using a deposited structure, PDB entry 3cw5 (Barraud *et al.*, 2008 ▶), consisting of 77 nucleotides. Experimental data were available to 3.1 Å resolution. Synthetic phases were created by starting from the refined structure and copying figures of merit (FOMs) from an experimentally phased structure, preserving the resolution and magnitude dependence of the FOMs. The mean FOM for the data to 3.1 Å resolution was 0.58. A random phase error was then selected from a distribution consistent with the FOM for that reflection and added to the phase. The performance of each search target was evaluated using the electron-density map calculated using the resulting phases and FOMs.

**Figure 2:**
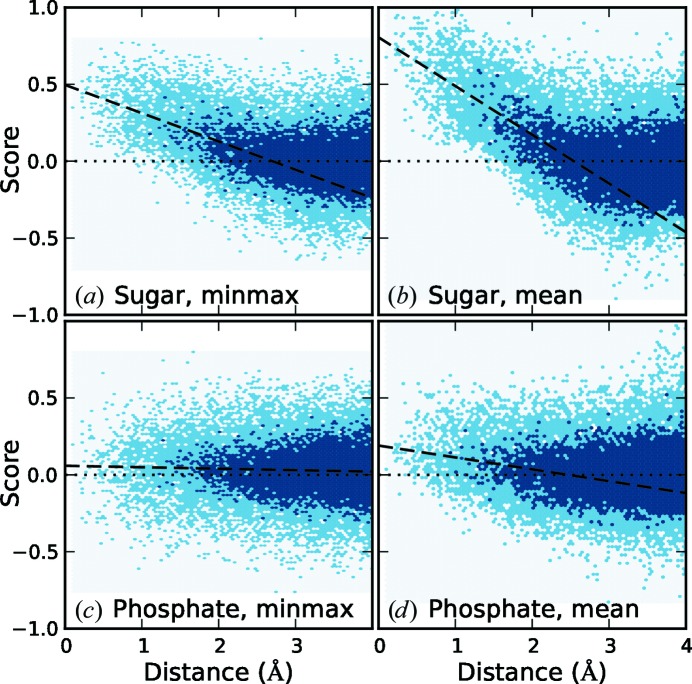
Comparison of the value of the search target score as a function of translational coordinate error for the correctly oriented sugar and phosphate targets, using either the *s*_minmax_ or *s*_mean_ scoring functions. The shading represents a density histogram of counts, with light shading for 1–4 or dark shading for 5+ counts per bin. Dashed lines show the regression of score on distance for distances less than 2.5 Å.

For each sugar or phosphate in the test structure, the search target was evaluated using the correct orientation and translations spanning a sphere of radius 4 Å around the true position. The values of *s*_minmax_ and *s*_mean_ were plotted against distance from the true position for each target, and are shown in Fig. 2 ▶.

The sugar search target shows significant discrimination using either the *s*_minmax_ or *s*_mean_ functions. The mean function is more effective in locating sugars; however, as discussed, it is less amenable to optimization.

The phosphate search target is less effective, with *s*_minmax_ showing very little signal. The mean function provides a weak signal, but also starts producing false positives beyond 2.5 Å from the true position as the target function begins to pick up tetrahedral C atoms in the sugar ring.

The poor performance of the phosphate target is in contrast to that of Keating & Pyle (2012 ▶), in which the phosphates form a starting point for building. The difference arises from the form of the search functions. The phosphate is distinguishable by the higher density of the P atom; however, *s*_minmax_ only reflects the lowest density at any of the high probe points and thus is blind to the phosphorus density. Similarly, in *s*_mean_ the phosphorus density is diluted by averaging over the high probe points. As a result, the phosphate target is primarily of value when used in conjunction with the sugar target.

### 3.2. Model building   

The method described here is optimized for interactive use, and so does not incorporate all of the model-completion steps or the time-consuming refinement step employed in automated model-building calculations such as *ARP*/*wARP* (Langer *et al.*, 2008 ▶) or *phenix.autobuild* (Terwilliger *et al.*, 2008 ▶). However, it is interesting to see how much of the model can be built in a single step with no refinement or improvement of the electron-density map.

35 nucleotide structures from the PDB for which experimental observations were available were used as test cases. For each structure, a simulated experimental data set was created using the same method as in §[Sec sec3.1]3.1. The resulting maps were used as a basis for finding search fragments and growing them into chains. The search algorithm was modified to search the whole asymmetric unit, rather than just a region around the current view centre.

**Figure 3:**
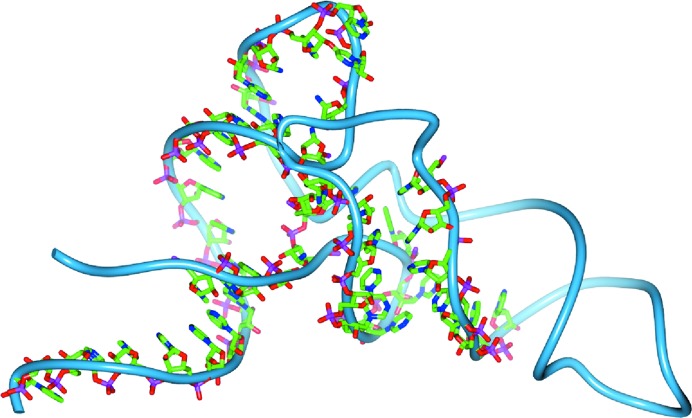
Comparison of autobuilt nucleotides with the final structure for 3cw5 at 3.1 Å resolution. The backbone trace of the full structure is show using a worm representation, and backbone atoms for the autobuilt model are shown as bonds.

The resulting partial models were then compared against the deposited structures and scored on the basis of the number of C1′ atoms in the deposited structure for which a C1′ atom was present in the partial model within 1.5 Å of the refined position. The results are shown in Table 1 ▶. Predominantly helical structures tend to be built almost completely. More complex folds are partially built, with some common hairpins being recognized. In some cases unbuilt regions are characterized by poor density, while in others they are regions of less common conformations. The deposited structure and the autobuilt model for the structure 3cw5 are shown in Fig. 3 ▶. In this case about half of the structure has been built.

The test structures were also used to judge the utility of the phosphate target. Omitting the phosphate target from either the search or the growing steps decreases the total proportion of the structures built, although in the case of the growing step the difference is marginal.

## 4. Discussion   

One notable result of this work is that fitting nucleotides appears to be a simpler problem than fitting proteins. This was initially surprising, given that nucleotides have greater torsional variability in the main chain (Murray *et al.*, 2003 ▶) and typically higher thermal displacement parameters (Keating & Pyle, 2010 ▶), leading in turn to greater ambiguity in the features of the electron-density map. However, the rigid groups within each nucleotide are larger and the most common relationships between neighbouring nucleotides show less variation than might be expected from the torsional variability for individual bonds. Attempts to apply the same kind of techniques to proteins has not yielded comparable benefits except in the case of large secondary-structure features (Langer *et al.*, 2006 ▶).

When applied to automated model building and refinement, the methods developed here are sufficiently fast that refinement becomes the rate-limiting step. The implementation of high-performance computing methods for crystallography (Sauter *et al.*, 2013 ▶) might in future increase the speed of the refinement step, which would be of immediate value to comprehensive ligand searches and some molecular-replacement problems; however, with the developments described here the building of novel nucleotide structures may also benefit.

There is scope for further applications of fingerprint techniques. The determination of the high and low probe points may be automated either using *ad hoc* rules to emulate the manual approach adopted here, or through a more rigorous information theoretical approach. These are being explored in the context of carbohydrate model building, but should also be applicable to the location of rigid or semi-rigid ligands.

### 4.1. Software availability   

The software is available as a part of the *Coot* model-building package from version 0.7 using the scripting command find_nucleic_acids_local(6) or by a right mouse click on the top tool bar to add the ‘NA build’ button. From build 5199 it is also available through the ‘Other Modelling Tools’ menu. A slower implementation which includes model refinement is available in the *CCP*4 software suite from version 6.3.0 under the name *Nautilus*. 

## Supplementary Material

PDB files and electron-density maps for the search targets.. DOI: 10.1107/S2052252514019290/fc5003sup1.zip

## Figures and Tables

**Table 1:** RNA test structures along with the proportion built in a single cycle of building without refinement or map recalculation Four structures (2g3s, 3c3z, 3f2q and 406d) contain reversed chain fragments leading to inflated phosphorus coordinate errors. The remaining large errors are typically indicative of a single fragment built inside out or jumping across chains.

		Nucleotides			
Structure	Resolution ()	Built	Total	Fraction (%)	Phosphorus error ()
157d	1.84	21	22	95	0.56
1d4r	2.00	61	82	74	0.86
1j6s	1.40	0	19	0	
1kd5	1.58	12	20	60	0.87
1kh6	2.72	19	46	41	0.83
1q96	1.75	64	78	82	0.97
1t0e	1.70	25	33	75	0.79
1u9s	2.90	35	155	22	0.79
1y26	2.10	29	70	41	1.46
1z7f	1.99	39	45	86	0.78
2a0p	1.95	14	14	10	0.46
2a64	3.30	66	292	22	1.24
2fd0	1.80	31	44	70	0.51
2g3s	1.50	17	70	24	1.70
2h1m	2.89	5	30	16	0.74
2oe5	1.50	27	31	87	0.64
2pn4	2.32	23	84	27	0.75
2r1s	1.41	14	24	58	0.85
2r22	1.50	0	24	0	
2v6w	1.81	12	12	100	0.49
2z75	1.70	72	142	50	0.90
359d	2.90	26	39	66	0.94
398d	1.94	26	28	92	0.66
3b31	2.35	30	42	71	0.81
3c3z	1.49	35	44	79	1.54
3cw5	3.10	36	77	46	1.29
3d2v	1.97	50	154	32	1.17
3e5c	2.25	24	52	46	0.87
3f2q	2.95	30	109	27	1.73
3fs0	2.20	17	19	89	0.65
3gm7	1.58	29	34	85	0.60
3k1v	2.20	17	27	62	1.41
406d	1.80	9	32	28	2.23
439d	1.60	14	14	100	0.45
466d	1.16	12	24	50	0.50
